# DEVELOPMENT OF THE ITALIAN VERSION OF THE MODIFIED BARTHEL INDEX AND PRELIMINARY RELIABILITY IN ADULTS WITH STROKE

**DOI:** 10.2340/jrm.v57.44279

**Published:** 2025-09-30

**Authors:** STEFANO DORONZIO, DIEGO LONGO, MICHELE PIAZZINI, ANGELA M. POLITI, TOMMASO CIAPETTI, MONICA BARNABE, CHIARA CASTAGNOLI, DONATA BARDI, JULIETA GIACANI, MARIA L. DEL VICARIO, GIULIO CHERUBINI, MARCO BACCINI, FRANCESCA CECCHI

**Affiliations:** 1 Department of Experimental and Clinical Medicine, University of Florence, Florence; 2 Neuromotor Research Unit, IRCCS Fondazione Don Carlo Gnocchi, Florence, Italy

**Keywords:** stroke, neurological rehabilitation, cross-cultural adaptation, reliability, disability assessment

## Abstract

**Objective:**

This study aimed to develop the Italian version of the modified Barthel Index and assess its reliability within stroke survivors.

**Design:**

Single-centre, prospective observational study for measure validation.

**Subjects/Patients:**

To pre-test the pre-final scale, stroke survivors, caregivers, and health professionals were enrolled. For reliability, only stroke survivors were enrolled. Inclusion of stroke survivors: adults presenting ADL limitation; exclusion: severe visual/hearing impairment, Mini-Mental State Examination <21; severe language disorder, clinical instability.

**Methods:**

The Italian modified Barthel Index was developed through forward-and-back translation, and interdisciplinary review. Clarity was evaluated by a sample of all participants. Inter-rater reliability was assessed by 2 independent physiotherapists, and test–retest examined 1–3 days later. Intraclass correlation coefficient (ICC), Spearman’s correlation, quadratic weighted Kappa, and agreement percentages were calculated.

**Results:**

Clarity was evaluated with 30 participants (10 per group); minor semantic adjustments were made. Reliability was assessed within 51 stroke survivors, showing Spearman’s correlation of 0.990 (test–retest) and 0.985 (inter-rater). ICCs were 0.990 and 0.987, respectively. Weighted Kappa values ranged from 0.76 to 0.98.

**Conclusion:**

The Italian Modified Barthel Index showed high reliability, supporting its use in Italian-speaking stroke populations. The validation of its other psychometric properties needs further research.

## LAY ABSTRACT

The Barthel Index is a commonly used clinical scale to assess how independently a person can perform daily activities after a stroke. Its modified version, the modified Barthel Index, offers improved clinical value but had no validated Italian version. In this study, we developed the Italian modified Barthel Index through a rigorous translation and review process. Thirty participants, including stroke survivors, caregivers, and healthcare professionals, assessed the clarity of the translation, leading to minor adjustments. We then tested its reliability, the degree to which assessments by the same rater at different times or by different examiners, is consistent, in 51 stroke survivors. Results showed excellent consistency between different examiners and across repeated assessments, with high correlation and agreement scores. These findings support the Italian modified Barthel Index as a reliable tool for evaluating independence in daily activities among Italian-speaking stroke survivors. Further studies are needed to explore other measurement properties.

Independence in activities of daily living (ADLs) can be defined as how a person functions independently in daily activities such as self-care and mobility (1). A reliable, valid, and sensitive assessment of independence in ADLs is a keystone to establishing care needs, setting rehabilitation goals, and evaluating outcomes.

The Barthel Index (BI) (2) is a worldwide broadly adopted tool in various medical conditions (3–6). It investigates independence in feeding, bathing, grooming, dressing, bowel control, bladder control, toileting, chair transfer, ambulation, and stair climbing. Items are scored on a scale of 2, 3, or 4 levels, in 5-point increments. The total score ranges from 0–100, where lower scores indicate higher dependence. An alternative scoring system with 1-point increments and total score ranging from 0–20 has been proposed by Collin et al. (7), who also reordered the 10 items and clarified the instructions. The BI is quickly administrable, and was found to be reliable (8), and valid (9) in stroke survivors.

To improve sensitivity, Shah et al. (10) proposed a modified version (the modified Barthel Index, mBI), introducing a 5-level scoring system for all items, while maintaining a total score up to 100 points. The authors tested the mBI in a sample of 258 participants with stroke outcomes and found that it has higher responsiveness than the original BI (10). This result has recently been confirmed in a similar population (11).

Both the original BI and the mBI have been cross-culturally adapted among various countries and languages (12–18). However, while an Italian version of the BI has been published (19), the mBI has not yet been cross-culturally adapted and tested in Italian, even if unofficial versions circulate in clinical practice.

To ensure equivalence in the translation of a clinical scale, a cross-cultural adaptation process is needed to address both language (translation) and cultural adaptation issues (20–22). Cross-cultural adaptation ensures the retention of psychometric properties such as validity and reliability, allowing comparability of data among different cultures (23). The Stroke Recovery and Rehabilitation Roundtable has recently emphasized the importance of using standardized agreed assessments with strong metric properties and clinical utility (24). Moreover, the Italian Society of Physical and Rehabilitation Medicine has recently confirmed the mBI as 1 of the essential tools to administer to stroke survivors for a complete and uniform assessment in Italy (25). Therefore, as the need for an Italian cross-culturally adapted version of the mBI is stronger than ever, this study aimed to (*i*) develop the Italian version of the mBI (mBI-IT) by a rigorous adaptation process, and (*ii*) preliminarily test its reliability.

## METHODS

### Study design, setting, and registration

This was a single-centre prospective observational study carried out at the Fondazione Don Carlo Gnocchi ONLUS (FDG), Florence, Italy. The study was split into 2 phases, i.e., translation of the mBI into Italian, including the pre-testing of the translation to evaluate its clarity and develop the final mBI-IT (phase 1) and the preliminary evaluation of its reliability (phase 2). The study was registered on ClinicalTrials.gov with the registration number NCT05831215. On 31 May 2023, it was approved by the local Ethics Committee CEAVC with the registry number 22257_oss.

### Phase 1: Cross-cultural adaptation process

*Development of the Italian modified Barthel Index (mBI-IT).* To develop the mBI-IT, the multi-step procedure described in Sousa and Rojjanasrirat (22) was followed. Along with the scale, the whole users’ instructions, including items and scoring description, were translated. Two certified translators independently completed the forward translation into Italian of the mBI. A multidisciplinary expert team, composed of physiatrists, physiotherapists, an occupational therapist, and a psychologist from the Neuromotor Research Unit of FDG compared and reviewed the 2 versions to produce a preliminary mBI-IT. This version was then independently back-translated into English by 2 different certified translators, who were blind to the original mBI. Last, the multidisciplinary team, joined by all the translators involved, reviewed and compared the 2 back-translated versions with the original scale, to ensure the conceptual, semantic, and operational equivalence of the translated scale. After the revision, the pre-final mBI-IT was produced.

The clarity of the pre-final version of the scale was assessed by enrolling a sample of users, comprising 10 stroke survivors, 10 caregivers of stroke patients, and 10 health professionals (physicians and physiotherapists), who agreed to participate in the study by signing informed consent.

For stroke survivors, inclusion criteria were: age >18 years old; impacted independence in ADLs. Exclusion criteria were: cognitive impairment defined by a Mini-Mental State Examination (MMSE) <21 (26); severe and uncorrectable visual and/or hearing impairment; severe language disorder that prevented understanding and execution of protocol procedures; and clinical instability, defined by a Clinical Instability Scale score >0, with a score of 0 indicating a low likelihood of short-term deterioration based on recognized medical standards (27).

For caregivers, inclusion criteria were: age >18 years old; being a part- or full-time caregiver (also a family member) of a stroke survivor with functional dependency in ADLs; willingness for study participation; and signing of the consent form. Exclusion criteria were: severe and uncorrectable visual and/or hearing impairment; and MMSE <21 (26).

For health professionals, inclusion criteria were: being a medical doctor, an occupational therapist, or a physiotherapist with at least 3 years of experience in the functional assessment of subjects with stroke; willingness for study participation; and signing of the consent form.

All participants were required to carefully read the pre-final version of the scale and indicate any unclear points in the instructions, the items, and the scoring points of the scale. They were asked to judge each mBI-IT item (including scoring instructions) as “Clear” or “Unclear” and the percentage of “Unclear” ratings was calculated. When it exceeded 20%, the item was revised by the multidisciplinary team to produce the final mBI-IT.

### Phase 2: Reliability assessment

The reliability of the final mBI-IT was assessed in a sample of patients enrolled according to the same criteria described above, among persons referred from acute-care hospitals to the FDG Neuromotor Rehabilitation Unit for rehabilitation after a stroke. Patients were consecutively enrolled, provided they met the eligibility criteria and signed the informed consent, until the data collection of more than 50 participants was completed. This sample size was chosen in order to reach an “adequate” sample, according to the COSMIN (Consensus-Based Standards for the Selection of Health Measurement Instruments) guidelines criteria (28, 29).

Pairs of raters, drawn randomly from a group of 10 experienced physiotherapists, were consecutively assigned to patients as they were enrolled. The 2 raters independently administered the scale to the assigned participants at an interval of about 1 h from each other. One of them (the first extract when pairs were formed) administered the mBI-IT again after 1–3 days. The short interval between the test and retest evaluations was chosen considering that the participants were all in a subacute poststroke phase, where even rapid changes are expected, so as to minimize possible differences in their functional level between the 2 evaluations. Raters were always blind to the other rater’s assessment and to their own prior assessment.

All items were evaluated by directly observing the patient at the bedside, except for the 3 items (bladder control, bowel control, and bathing) for which the assessment was made by asking the hospital staff. Before starting data collection, raters participated in 2 x 1-hour training sessions to align in test administration and scoring.

### Data analysis

For test–retest and inter-rater reliability, repeated assessments of the same rater and assessments of Rater 1 and Rater 2 were compared, respectively. For the total mBI-IT scores, the distribution of the data was preliminarily evaluated by means of the Shapiro–Wilk test, which showed that not all scores fitted into a normal distribution (*p*<0.05). Hence, the Wilcoxon test was applied to examine any significant differences in these scores between the 2 assessment sessions. For the total mBI scores, the 1-way random ANOVA Intraclass Correlation Coefficient for single measures (ICC1,1) and Spearman’s correlation coefficient were computed. For scores of each mBI-IT item, quadratic weighted Kappa (wK) and percentages of absolute agreement were computed. For the interpretation of Spearman’s rho and ICC, we adopted the criteria proposed by Fitzpatrick et al. (30), who set different standards for using the scale at group level (correlation coefficient of at least 0.70) or at individual level (correlation coefficient of 0.70 or at least 0.90, respectively). As for kappa coefficients, we interpreted the agreement as suggested by McHugh (31): 0–0.20=none; 0.21–0.39=minimal; 0.40–0.59=weak; 0.60–0.79=moderate; 0.80–0.90=strong; >0.90=almost perfect. As the paired differences (RETEST score–TEST score, or score of Rater 2–score of Rater 1) were also not normally distributed, the error of measurement of the total score was assessed by computing the non-parametric (97.5th and 2.5th percentile) Bland Altman Limits of Agreement, both absolute (LOA) and as a percentage (LOA%=LOA/grand median x 100), and the median of the differences as average bias (32). For the interpretation of the LOAs, the criteria commonly reported in the literature for Minimal Detectable Change were used, i.e., excellent if less than 10% and acceptable if between 10% and 30% (33,34). The statistical analysis was performed with the Jamovi (version 2.5) computer software (The jamovi project, 2024; https://www.jamovi.org).

## RESULTS

### Phase 1: Translation

The word “patient” was always translated as “persona” (“person”), in agreement with current recommendations to remove the word “patient” from the clinical vocabulary to see beyond diseases, acknowledge the unique individual, and highlight that prescriptions and support by healthcare providers should always take into consideration the person’s needs, values, and desires (35). Moreover, a few locutions required a deviation from the literal translation and the achievement of a consensus by the multidisciplinary team:

- “Wheelchair management” was translated with “spostamenti in carrozzina” (i.e., moving around in a wheelchair);
- “Stairs”, “climb stair”, “stair climbing”, and “ascend/descend” were always translated as “salire/scendere le scale” (i.e., ascending/descending stairs);
- “On and off the toilet” was translated as “uso del gabinetto” (i.e., use of the toilet);
- “Bowels” and “Bladder”/**“**bladder management” were translated as “Controllo dell’intestino” (bowel control) and “Controllo della vescica” (bladder control), respectively.

### Pre-test of the pre-final mBI-IT

Ten persons with stroke (7 men, age=61.4±16.6), 10 caregivers (4 men, age=58±15.43), and 10 health professionals (2 men, age=41.3±11.59) evaluated the clarity of the pre-final mBI-IT (Table I). Only scoring instructions for the items “ambulation” and “toilet” were found to be unclear by more than 20% of participants, who could hardly distinguish between the scoring levels 1 and 2.

**Table I:** Clearness percentage of the Italian modified Barthel Index pre-final version

Item	Item clearness	Score I clearness	Score 2 clearness	Score 3 clearness	Score 4 clearness	Score 5 clearness	Notes clearness
Instructions	92.22%	90.00%	93.33%	93.33%	90.00%	96.67%	90.00%
1: Trasferimenti sedia/letto	92.67%	96.67%	93.33%	83.33%	96.67%	93.33%	–
2a: Deambulazione	88.00%	73.33%*	100.00%	90.00%	83.33%	93.33%	–
2b: Spostamenti in carrozzina	93.33%	93.33%	86.67%	96.67%	90.00%	100.00%	93.33%
3: Scale	99.33%	100.00%	100.00%	96.67%	100.00%	100.00%	–
4: Uso del gabinetto	92.00%	76.67%*	86.67%	100.00%	96.67%	100.00%	–
5: Controllo dell’intestino	95.33%	100.00%	90.00%	96.67%	96.67%	93.33%	–
6: Controllo della vescica	93.33%	93.33%	83.33%	96.67%	96.67%	96.67%	–
7: Fare il bagno	92.67%	90.00%	93.33%	90.00%	96.67%	93.33%	–
8: Vestirsi	91.33%	96.67%	93.33%	80.00%	96.67%	90.00%	–
9: Igiene personale	96.67%	100.00%	90.00%	93.33%	100.00%	100.00%	–
10: Alimentazione	91.33%	93.33%	93.33%	96.67%	90.00%	83.33%	–

1: Chair/Bed Transfers; 2: Ambulation; 2b: Ambulation/Wheelchair; 3: Stair Climbing; 4: Toilet Transfers; 5: Bowel Control; 6: Bladder Control; 7: Bathing; 8: Dressing; 9: Personal Hygiene; 10: Feeding.

- *Ambulation:* indeed, both scoring levels 1 and 2 were assigned when the patient was not able to walk on their own and a clear difference between the 2 points was lacking. Thus, we replaced scoring level 1, i.e., “La persona è dipendente nella deambulazione” (the person is dependent in ambulation) with “La persona non è in grado di deambulare” (the person is not able to ambulate).
- *Toilet:* in this case too, both scoring levels are assigned when the patient is not able to manage different aspects of toileting by him/herself. To note the difference between the 2 levels, we replaced the original description of level 1, i.e., “La persona è completamente dipendente nell’uso del gabinetto” (the person is completely dependent in the use of the toilet) with “la persona non è in grado di collaborare in nessuna fase dell’uso del gabinetto” (the person is unable to cooperate at any stage of toilet use). The final mBI-IT is available as Appendix S1.

### Phase 2: Preliminary reliability assessment

We screened for eligibility 83 consecutive persons with stroke admitted to the subacute neuromotor rehabilitation unit of FDG; 32 patients did not meet the inclusion criteria because of clinical instability (6), cognitive deficits (5), refused informed consent (19), language barrier (1), and lower limb amputation (1). All included subjects completed the assessment procedures, resulting in a sample of 51 subjects for both test–retest reliability and inter-rater reliability (Table II). The 10 raters, with at least 2 years of experience in stroke rehabilitation and specific training in administering the scale, performed on average 5.1±2.6 (range: 3–12) assessments in total, of which 5.1±2.8 (range: 3–12) by Rater 1 and 5.1±2.6 (range: 3–10) by Rater 2. Thus, all 10 raters participated in both the test–retest and inter-rater assessment for some participants. The retest was conducted on average 1.76±0.93 days after the test, with 3 participants reassessed after 4 day for contingency reasons.

**Table II:** Clinical characteristics of stroke survivors in Phase II

Variable	Value
Sex, n (%)	
Men	30 (58.8%)
Women	21 (41.2%)
Age (years), mean (SD), range	74.3 (9.65), 48–93
More affected side, n (%)	
Left	21 (41.2%)
Right	28 (54.9%)
Both	2 (3.9%)
Time since stroke (days), mean (SD), range	28.4 (18.2). 10–96
MMSE, mean (SD), range	24.36 (5.70), 12–30
mRS, mean (SD), range	3.73 (0.69), 1–5
NIHSS, mean (SD), range	6.74 (4.61), 0–20
FMA motor function, mean (SD), range	64.05 (30.66), 8–100

SD: standard deviation; MMSE: Mini Mental State Examination; mRS: modified Rankin Scale; NIHSS: National Institute Health Stroke Scale; FMA: Fugl-Meyer Assessment Scale.

**Fig. 1:**
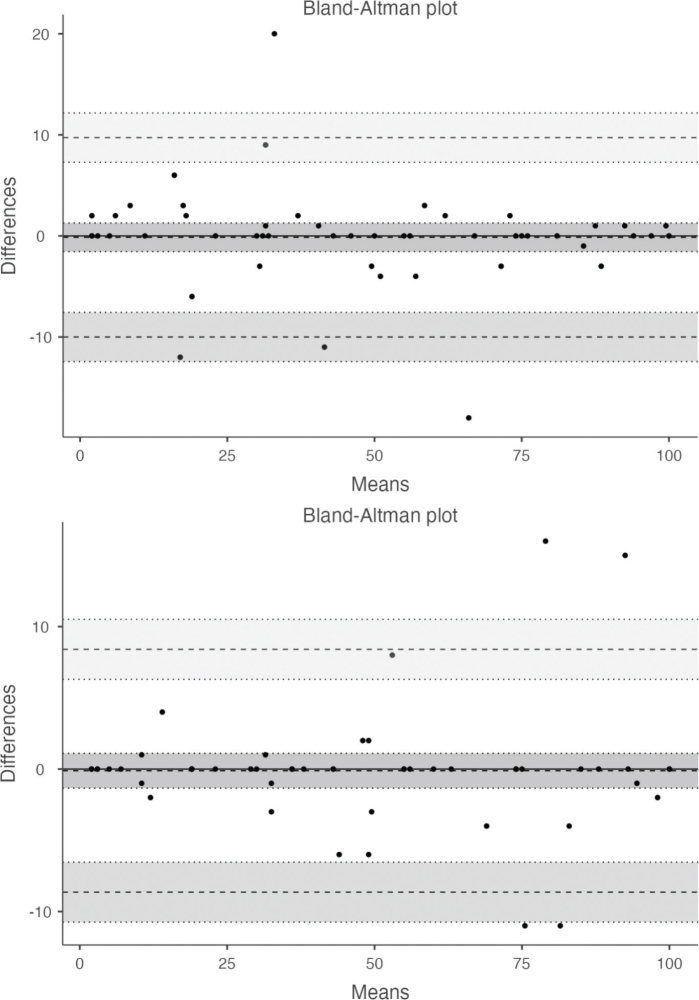
Bland–Altman graph for both test–retest and inter-rater evaluations.

As for the total score, no significant differences between assessments were found, and a Spearman’s rho of 0.990 and 0.985, and an ICC of 0.990 and 0.987, were computed for test–retest and inter-rater reliability, respectively (Table III). The LOA ranged from –8.6 (18.5%) to +8.4 (18%), and from –10 (26.6%) to +9.7 (20%), for the test–retest and the inter-rater measurement error, respectively (Fig. 1). For individual item scores, the wK and the OA ranged from 0.76–0.98 and 0.76–0.96 (test–retest, Table IV), and 0.72–0.95 and 0.76–0.94 (inter-rater, Table V), respectively.

**Table III:** Test–retest and inter-rater reliability of Italian modified Barthel Index scores

Item	Test	Retest	*p*-value*	ICC (95%CI)	rho	Upper LOA (%)	Lower LOA (%)	Median of difference
	Median (IQR), min–max	Median (IQR), min–max						
Test–retest	46 (53), 2–100	47 (53), 2–100	0.423	0.990 (0.984–0.994)	0.990	8.4 (18)	–8.6 (18.5)	0
Inter-rater	46 (53), 2–100	47 (51.5), 1–100	0.900	0.987 (0.979–0.992)	0.985	9.7 (20)	–10 (26.6)	0

IQR: interquartile range; Min: minimum; Max: maximum; ICC: Intraclass Correlation Coefficient; rho: Spearman’s correlation coefficient; LOA: limit of agreement.

* Wilcoxon test.

**Table IV:** Test–retest reliability of individual Italian modified Barthel Index item scores

	Assessment 1 Median (IQR), min–max	Assessment 2 Median (IQR), min–max	OA	wK (95% CI)
Chair/bed transfer	8 (9), 0–15	8 (9), 0–15	0.96	0.889 (0.740–1.000)
Ambulation	3 (12), 0–15	3 (12), 0–15	0.88	0.862 (0.727–0.997)
Wheelchair*	0 (1), 0–5	0 (0), 0–5	0.91	0.914 (0.792–1.000)
Stair climbing	0 (2), 0–10	0 (2), 0–10	0.92	0.836 (0.668–1.000)
Toilet transfers	5 (8), 0–10	5 (8), 0–10	0.84	0.855 (0.734–0.976)
Bowel control	8 (8), 0–10	8 (8), 0–10	0.94	0.982 (0.963–1.000)
Bladder control	8 (10), 0–10	8 (10), 0–10	0.92	0.810 (0.606–1.000)
Bathing	1 (3), 0–5	1 (3), 0–5	0.90	0.937 (0.874–1.000)
Dressing	5 (6), 0–10	5 (6), 0–10	0.86	0.762 (0.573–0.951)
Personal hygiene	4 (4), 0–5	4 (4), 0–5	0.92	0.965 (0.922–1.000)
Feeding	8 (5), 0–10	8 (5), 0–10	0.94	0.935 (0.832–1.000)

wK: weighted kappa (squared); OA: percentage of observed agreement.

* If unable to walk.

**Table V:** Inter-rater reliability of individual Italian modified Barthel Index item scores

Item	Assessment 1 Median (IQR), min–max	Assessment 2 Median (IQR), min–max	OA	wK (95% CI)
Chair/bed transfer	8 (9), 0–15	8 (9), 0–15	0.84	0.774 (0.584–0.964)
Ambulation	3 (12), 0–15	3 (8), 0–15	0.88	0.804 (0.644–0.964)
Wheelchair*	0 (1), 0–5	0 (0), 0–3	0.94	0.900 (0.744–1.000)
Stair climbing	0 (2), 0–10	0 (5), 0–10	0.88	0.887 (0.765–1.000)
Toilet transfers	5 (8), 0–10	5 (8), 0–10	0.84	0.879 (0.772–0.987)
Bowel control	8 (8), 0–10	8 (8), 0–10	0.88	0.780 (0.581–0.978)
Bladder control	8 (10), 0–10	8 (10), 0–10	0.84	0.723 (0.533–0.912)
Bathing	1 (3), 0–5	1 (3), 0–5	0.84	0.952 (0.920–0.983)
Dressing	5 (6), 0–10	5 (6), 0–10	0.86	0.849 (0.709–0.988)
Personal hygiene	4 (4), 0–5	4 (4), 0–5	0.76	0.925 (0.874–0.977)
Feeding	8 (5), 0–10	8 (5), 0–10	0.88	0.914 (0.806–1.000)

wK: weighted kappa (squared); OA: percentage of observed agreement.

* If unable to walk.

## DISCUSSION

The present study developed the mBI-IT by a rigorous process of transcultural adaptation and pilot-testing in a sample of health professionals, caregivers, and stroke patients, and provided a comprehensive test–retest and inter-rater reliability analysis for both the total score and for the individual item scores.

The final mBI-IT showed excellent reliability, with no systematic bias between the 2 assessments under comparison, confirming that the increased scoring partition does not affect the reliability of the scale. In fact, our results are quite similar and even better than those reported for the Italian version of the original BI, i.e., ICC=0.983 for the test–retest reliability (19) and ICC=0.960 for the inter-rater reliability (3). Data presented show that the mBI-IT, like the Italian version of the BI, has sufficient reliability to also be used on an individual level (30), supporting the suitability of the scale for use in clinical settings. Yang et al. (36) compared the test–retest reliability of the English version of BI and mBI on 60 Taiwanese chronic stroke survivors, finding very similar results (ICC=0.94 for both BI and mBI, respectively).

Currently, the mBI has been officially translated into Chinese (14), Greek (16), Turkish (18), Iranian (37), Korean (17), and Japanese (38). Reliability data collected in stroke participants are not available for all versions; moreover, researchers employed different evaluators and calculated different reliability coefficients. Overall, our findings on test–retest reliability are in line with those reported for other versions of the scale, i.e., correlation coefficients of 0.990 (Japanese version, 30 participants assessed twice by 10 raters, physiotherapists or occupational therapists, who watched video recordings of participants performing different activities of daily life; mean ICC from all raters) (38), 0.937 (Korean version, 30 participants assessed twice by 15 raters, physiatrists or occupational therapists, mean Kendall’s coefficient of concordance from all raters) (17), and 0.996 (Greek version, 41 participants assessed twice by a single unspecified rater, 20 with stroke and 21 with spinal cord injury, ICC) (16). The inter-rater reliability has been investigated only for the Japanese (38), Turkish (50 participants with subacute stroke evaluated by 2 unspecified raters) (18), and Korean (17) versions, finding ICC=0.990, ICC=0.990, and Spearman’s rho=0.957 (mean coefficient calculated from values found in 5 groups of 3 raters), respectively.

An important index of reliability is the estimated measurement error (39), for which we computed non-parametric LOAs that can be considered acceptable but not negligible. The median of the differences between the scores of different raters or test and retest evaluations were close to zero, indicating the absence of systematic bias, but the random variability, plus or minus, was always between 10% and 30%. Unfortunately, the measurement error of the mBI has only been evaluated for the original English version (36) and the Greek version (16), in both cases limited to test–retest comparison. For the former, Yang et al. (36) reported a percentage MDC95 value quite similar to our LOA (19%), whereas for the Greek version Ferfeli et al. (16) calculated the parametric LoA and found significantly lower values. The authors of this work reported the values only in graphical form, and we extracted a value about 4 points from their graph (upper limit: 3.8; lower limit: –4.2), corresponding to a percentage of about 9% (upper limit: 8.4%; lower limit: 9,4%). However, in this study a single rater administered the mBI to all participants and, furthermore, no information is given on the characteristics of this rater; thus, we think that the results are hardly generalizable.

With regard to individual mBI items, we found substantial or almost perfect agreement in 10/11 items (test–retest agreement) and 8/11 items (inter-rater agreement). Only for the item “dressing” (test–retest) and the items “Ambulation”, “Bowel control” and “Bladder control” (inter-rater) do kappa coefficients indicate moderate agreement. The reliability of the mBI items has so far only been estimated for the Chinese (14) (limited to inter-rater) and the Japanese (both inter- and intra-rater) (38) versions, for which slightly better results were reported. In these 2 studies, kappa coefficients consistently above 0.80 for all items were found, except for the items “Personal hygiene” and “Self-bathing” for the inter-rater agreement of the Japanese version, which showed moderate agreement according to our criteria. However, in both studies, assessors rated the same performance by watching video recordings (38), or by independently assigning the mBI score while observing another assessor administering the scale (14), thus eliminating the possible source of variability due to minimal changes in the participant’s performance between assessments. Moreover, in the study on the Japanese version (38), the choice of videotaping participants during different ADLs led to a simplification of the tasks in some cases: for some items (self-bathing, toileting), participants were videotaped with their clothes on for privacy, but this excluded important tasks that must be fulfilled – for example, fasten and unfasten clothes, or prevent soiling of clothes; for bowel and bladder control, a nurse describing the patient’s capacity was recorded; for personal hygiene, only the task of tooth brushing was taped; and for dressing the tasks of putting on or removing orthoses were not recorded. Conceivably, all these features might easily explain the slightly different results compared with the present study.

### Study limitations

Our study has some limitations, the first concerning the generalizability of the results to health professionals other than occupational therapists or physiotherapists. Moreover, although participants included a wide range of functional levels, they were all in the subacute phase after stroke and we cannot be certain that the results can be applied to different phases after stroke or other disabling conditions. On the other hand, enrolling people with subacute stroke forced us to minimize the distance between test and retest, to avoid the occurrence of recovery-related functional changes between the 2 evaluations, but this increased the risk of a possible recall bias with an overestimation of reliability; we nevertheless believe that this should have been minimal, because raters typically administered many assessment scales and remembering the scores assigned to the different mBI items for a particular patient should have been difficult.

### Conclusions

In conclusion, the mBI-IT demonstrated good to excellent reliability, with results comparable to or even surpassing those of the Italian BI and other cross-cultural adaptations of the mBI. These results provide initial support for the use of the mBI-IT in Italian-speaking populations, but further studies are needed to verify other metric properties of the scale.

## Supplementary Material


