# Supplementary material for: DEVELOPMENT OF THE ITALIAN VERSION OF THE MODIFIED BARTHEL INDEX AND PRELIMINARY RELIABILITY IN ADULTS WITH STROKE
Source: J Rehabil Med. 2025 Sep 30;57:43279. doi: 10.2340/jrm.v57.44279 (PMC12495485; doi:10.2340/jrm.v57.44279)
Supplement: Supplementary file 1 [file JRM-57-44279-s1.pdf]

INDICE DI BARTHEL (IB) MODIFICATO (UFF)
Il punteggio modificato per l'IB segue lo stesso schema generale:<br>- Le persone che non sono in grado di eseguire l'attività sono codificate "1" sul modulo di valutazione; tale codifica contribuisce di zero punti al punteggio di Barthel totale;<br>- Le persone fortemente dipendenti e/o non sicure senza la presenza di qualcuno sono codificate "2";<br>- Le persone che richiedono assistenza e/o supervisione moderata per completare l'attività sono codificate "3";<br>-Le persone che richiedono assistenza e/o supervisione minima sono codificate "4";<br>- Le persone completamente indipendenti sono codificate "5".<br>La lentezza di un individuo nello svolgimento di un'attività non è valutata con un punteggio minore, se non è richiesta alcuna assistenza umana per una funzione.

Indice di Barthel modificato – Versione prefinale

Item	Punteggio	
TRASFERIMENTI SEDIA/LETTO	0	La persona non è in grado di partecipare a un trasferimento. Sono necessari due assistenti per trasferire la persona, con o senza dispositivo meccanico.
	3	La persona è in grado di partecipare ma è richiesta la massima assistenza di un'altra persona in tutti gli aspetti del trasferimento.
	8	Il trasferimento richiede l'assistenza di un'altra persona. L'assistenza può essere richiesta in un qualsiasi aspetto del trasferimento.
	12	La presenza di un'altra persona è richiesta o per rassicurare o per fornire supervisione per la sicurezza.
	15	La persona può avvicinarsi in sicurezza al letto camminando o su una carrozzina, bloccare i freni, sollevare i poggiapiedi o posizionare l'ausilio per il cammino, trasferirsi in sicurezza sul letto, sdraiarsi, mettersi seduto sul lato del letto, cambiare la posizione della carrozzina, trasferirsi di nuovo in carrozzina con sicurezza e/o afferrare l'ausilio e alzarsi in piedi. La persona deve essere indipendente in tutte le fasi di questa attività.

Item	Punteggio	
DEAMBULAZIONE	0	La persona non è in grado di deambulare.
	3	È richiesta la presenza costante di uno o più assistenti durante la deambulazione.
	8	È richiesta assistenza per raggiungere e/o maneggiare gli ausili. È richiesta una persona per fornire assistenza.
	12	La persona è indipendente nella deambulazione, ma non è in grado di camminare per 50 metri senza assistenza, oppure è necessaria supervisione per rassicurare o per la sicurezza in situazioni pericolose.
	15	La persona deve essere in grado di indossare i tutori, se richiesto, bloccare e sbloccare tali tutori, raggiungere la stazione eretta, sedersi e posizionare per l'uso gli ausili necessari. La persona deve essere in grado di usare stampelle, bastoni o un deambulatore (se necessari) e camminare per 50 metri senza aiuto o supervisione.

Item	Punteggio	
SPOSTAMENTI IN CARROZZINA (ALTERNATIVA A DEAMBULAZIONE)*	0	La persona è dipendente per gli spostamenti con la carrozzina.
	1	La persona può spingersi per brevi distanze su una superficie piana, ma è richiesta assistenza per tutte le altre fasi di gestione della carrozzina.
	3	È necessaria la presenza e l'assistenza costante di una persona per posizionare la carrozzina verso il tavolo, il letto, ecc..
	4	La persona può spingersi per una durata ragionevole su terreni che si incontrano regolarmente. Assistenza minima può essere ancora necessaria nelle curve strette.
	5	Per spingere la carrozzina in modo indipendente, la persona deve essere in grado di girare gli angoli, girare su se stessa, manovrare la carrozzina fino al tavolo, al letto, al water, ecc. La persona deve essere in grado di spingere la carrozzina per almeno 50 metri.
* Se non in grado di camminare<br>*Usare questa voce solo se al paziente è stato assegnato un punteggio 0 alla voce Deambulazione e solo se è stato allenato alla gestione della carrozzina.*		

Item	Punteggio	
SCALE	0	La persona non è in grado di salire/scendere le scale.
	2	È richiesta assistenza in tutti gli aspetti della salita/discesa delle scale, inclusa l'assistenza con gli ausili per il cammino
	5	La persona è in grado di salire/scendere ma non è in grado di trasportare gli ausili per la deambulazione e richiede supervisione e assistenza.
	8	Generalmente non è richiesta assistenza. A volte per la sicurezza è richiesta una supervisione, a causa di rigidità mattutina, mancanza di fiato, ecc.
	10	La persona è in grado di salire e scendere una rampa di scale con sicurezza senza aiuto o supervisione. La persona è in grado di usare corrimano, bastone o stampelle quando necessario e di trasportare questi ausili mentre sale o scende.

Item	Punteggio	
USO DEL GABINETTO	0	La persona è completamente dipendente nell'uso del gabinetto.
	2	È richiesta assistenza in tutti gli aspetti dell'uso del gabinetto.
	5	Può essere richiesta assistenza per la gestione degli indumenti, i trasferimenti o il lavaggio delle mani.
	8	Supervisione può essere richiesta per motivi di sicurezza per l'uso di un gabinetto normale. La persona può utilizzare una comoda durante la notte, ma è richiesta assistenza per lo svuotamento e la pulizia.
	10	La persona è in grado di sedersi/alzarsi dal water, allacciare e slacciare gli indumenti, evitare di macchiare gli indumenti e usare la carta igienica senza aiuto. Se necessario, la persona può usare una padella, un pappagallo o una comoda durante la notte, ma deve essere in grado di svuotarli e pulirli.

Item	Punteggio	
CONTROLLO DELL'INTESTINO	0	La persona ha incontinenza intestinale.
	2	La persona richiede aiuto per assumere la posizione appropriata e per le tecniche di facilitazione dell'evacuazione.
	5	La persona può assumere una posizione appropriata ma non è in grado di usare tecniche per facilitare l'evacuazione o pulirsi senza assistenza e ha frequenti episodi di incontinenza. È richiesta assistenza per utilizzare ausili per l'incontinenza come pannoloni, ecc.
	8	La persona può richiedere supervisione per l'uso di supposte o clisteri e ha occasionali episodi di incontinenza.
	10	La persona è in grado di controllare l'intestino e non ha episodi di incontinenza, può usare da solo una supposta o fare un clistere quando necessario.

Item	Punteggio	
CONTROLLO DELLA VESCICA	0	La persona è dipendente per il controllo vescicale, è incontinente o ha un catetere a dimora.
	2	La persona è incontinente, ma è in grado di collaborare nell'applicazione di dispositivi interni ed esterni.
	5	La persona è generalmente asciutta durante il giorno ma non di notte e ha bisogno di assistenza con i dispositivi.
	8	La persona è generalmente asciutta sia di giorno che di notte, ma può avere un occasionale episodio di incontinenza o necessitare di assistenza minima con i dispositivi interni o esterni.
	10	La persona è in grado di controllare la vescica sia di giorno che di notte e/o è indipendente con i dispositivi interni o esterni.

Item	Punteggio	
FARE IL BAGNO	0	La persona è completamente dipendente per fare il bagno.
	1	È richiesta assistenza in tutti gli aspetti del bagno.
	3	È richiesta assistenza per il trasferimento nella vasca/doccia, oppure per lavarsi o asciugarsi, inclusa l'incapacità di completare un'attività a causa di una condizione, una malattia, ecc.
	4	Per sicurezza è richiesta supervisione per la regolazione della temperatura dell'acqua o per il trasferimento.
	5	La persona è in grado di utilizzare la vasca, la doccia o fare una spugnatura completa. La persona deve essere in grado di eseguire tutte le fasi di qualsiasi metodo venga impiegato senza la presenza di un'altra persona.

Item	Punteggio	
VESTIRSI	0	La persona è dipendente in tutti gli aspetti del vestirsi e non è in grado di partecipare all'attività.
	2	La persona è in grado di partecipare in una certa misura, ma è dipendente in tutti gli aspetti del vestirsi
	5	È necessaria assistenza per indossare e/o togliere qualsiasi indumento
	8	È necessaria solo minima assistenza per allacciare indumenti come bottoni, cerniere, reggiseno, scarpe, ecc…
	10	La persona è in grado di indossare, allacciare e togliere gli indumenti, allacciarsi le scarpe, e indossare, allacciare e rimuovere corsetti e tutori, come prescritto

Item	Punteggio	
IGIENE PERSONALE	0	La persona non è in grado di occuparsi dell'igiene personale ed è dipendente in tutti gli aspetti.
	2	È richiesta assistenza in tutte le fasi dell'igiene personale.
	5	È richiesto un certo grado di assistenza in una o più fasi dell'igiene personale
	8	La persona è in grado di provvedere alla propria igiene personale, ma richiede minima assistenza prima e/o dopo le operazioni-
	10	La persona è in grado di lavarsi mani e viso, pettinarsi, pulirsi i denti e radersi. Una persona di sesso maschile può usare qualsiasi tipo di rasoio ma deve inserire la lama o collegare il rasoio senza aiuto, nonché recuperarlo dal cassetto o dall'armadietto. Una persona di sesso femminile deve truccarsi da sola, se usa il trucco, ma non necessariamente essere in grado di intrecciare o acconciare i capelli

Item	Punteggio	
ALIMENTAZIONE	0	La persona è dipendente per tutti gli aspetti e deve essere alimentata.
	2	La persona è in grado di manipolare uno strumento per mangiare, di solito un cucchiaio, ma qualcuno deve fornire assistenza attiva durante il pasto
	5	La persona è in grado di alimentarsi con supervisione. È richiesta assistenza in compiti associati come mettere latte/zucchero nel tè, sale, pepe, spalmare il burro, girare il piatto o in altre attività "di preparazione".
	8	La persona è indipendente nell'alimentazione con il vassoio preparato, ad eccezione eventualmente della necessità di tagliare la carne, aprire il cartone del latte o il coperchio di barattoli, ecc. Non è necessaria la presenza di un'altra persona.
	10	La persona è in grado di alimentarsi da un vassoio o da un tavolo, se qualcuno gli mette il cibo a portata di mano. Se necessario, la persona deve indossare un ausilio, tagliare il cibo e, se lo desidera, usare sale e pepe, spalmare il burro, ecc.
